# Magnetic Resonance Imaging Reveals Meningeal Lymphatic Impairment in Lung Adenocarcinoma Brain Metastasis Progression

**DOI:** 10.1002/advs.202516988

**Published:** 2026-01-15

**Authors:** Yuan Zhang, Shucheng Jin, Penghui Guo, Yuying Yin, Yichun Hua, Xiaosheng Ding, Zhe Zhang, Shuo Chen, Xu Han, Baowang Li, Yan Liu, Xiaoyan Li, Deling Li, Jing Jing, Wei Shi, Wang Jia

**Affiliations:** ^1^ Department of Neurosurgery Beijing Tiantan Hospital Capital Medical University Beijing China; ^2^ Beijing Neurosurgical Institute Beijing China; ^3^ Beijing Institute for Brain Research Chinese Academy of Medical Sciences & Peking Union Medical College Beijing China; ^4^ Chinese Institute for Brain Research Beijing China; ^5^ Clinical Trial Research Center Beijing Hospital National Center of Gerontology Institute of Geriatric Medicine Chinese Academy of Medical Sciences Beijing China; ^6^ Department of Oncology Beijing Tiantan Hospital Capital Medical University Beijing China; ^7^ Department of Neurology Beijing Tiantan Hospital Capital Medical University Beijing China; ^8^ Tiantan Neuroimaging Center of Excellence Beijing Tiantan Hospital Capital Medical University Beijing China; ^9^ China National Clinical Research Center For Neurological Diseases Beijing Tiantan Hospital Capital Medical University Beijing China; ^10^ Shanghai United Imaging Healthcare Co., Ltd Shanghai China; ^11^ Wuhan United Imaging Life Science Instrument Co., Ltd Wuhan China

**Keywords:** brain metastasis, cervical lymph nodes, disease progression, meningeal lymphatic vessels, MRI

## Abstract

Meningeal lymphatic vessels (mLVs) contribute to brain immune surveillance; however, their structural and functional alterations in brain metastasis remain incompletely defined. Here, we optimized a magnetic resonance imaging protocol to quantitatively analyze mLV structure and drainage function. Using murine lung adenocarcinoma brain metastasis models and clinical imaging data, we reveal significant mLV disruption surrounding the superior sagittal sinus (SSS), which results in impaired drainage to deep cervical lymph nodes (dCLNs). Among ten cervical lymph‐node levels assessed clinically, level IIA lymph nodes most accurately reflect mLV drainage efficiency. Functionally, mLV ablation compromised intrathecal chemotherapy efficacy in mice, while clinically, greater mLV structural and functional impairment correlates with disease progression in lung adenocarcinoma brain metastasis. Collectively, our findings demonstrate that lung adenocarcinoma brain metastases disrupt SSS‐adjacent mLVs (mLVs‐SSS), which potentially facilitates metastatic progression through compromised immune surveillance.

## Graphical abstract

This study demonstrates meningeal lymphatic impairment associated with lung adenocarcinoma brain metastasis progression in both human patients and mouse models, establishes clinically applicable non‐invasive imaging biomarkers for quantitative analyzing mLV structure and function, and reveals mLVs as robust prognostic indicators and promising potential therapeutic targets for effective brain metastasis management.

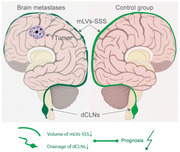

## 1 Introduction

Although the central nervous system (CNS) has long been considered an immune‐privileged site [1], innate and adaptive immune cells within the meninges actively monitor and respond to pathological conditions [2]. Notably, meningeal lymphatic vessels (mLVs) in the dura mater of both humans and mice facilitate the drainage of interstitial fluid and cerebrospinal fluid (CSF) to the deep cervical lymph nodes (dCLNs) [3, 4, 5], which supports waste clearance, immune cell trafficking, and macromolecule transport [6]. Recent studies have demonstrated that mLVs play an important role in drainage and immunosurveillance in brain tumors [7, 8]. Specifically, mLVs in patients with intracranial malignant tumors exhibit increased wash‐in but reduced wash‐out functionality [9], which suggests potential dysfunction in lymphatic drainage. However, it remains unclear if these functional drainage alterations are associated with structural abnormalities in mLVs among brain tumor patients and whether tumor‐induced mLV dysfunction further exacerbates brain tumor progression.

Magnetic resonance imaging (MRI) enables non‐invasive visualization and quantification of mLV structure and drainage in humans [10], and has been used in studies of aging [11, 12], Parkinson's disease [13], neuromyelitis optica spectrum disorders [14], chronic migraine [15], and brain tumors [9]. Techniques such as 3D T2‐fluid‐attenuated inversion recovery (FLAIR) imaging and T1‐weighted black‐blood (BB) imaging post‐gadobutrol injection are commonly used for semi‐quantitative mLV assessment [16, 17], while dynamic contrast‐enhanced (DCE)‐MRI evaluates drainage in mLVs and cervical lymph nodes (CLNs) [13, 14]. Despite these advances, detecting mLV changes in humans using current MRI sequences remains challenging. For example, although studies in laboratory mice using direct immunofluorescence staining reveal significant mLVs regress with age [18], human MRI studies using contrast‐enhanced 3T T1‐weighted BB sequences report an age‐associated increase in peri‐sinus dural lymphatic space volume [11]. This discrepancy suggests potential limitations in the sensitivity and accuracy of existing MRI sequences for quantitative mLV assessment, and underscores the need for optimized imaging protocols to improve precision and reliability.

In this study, we optimized a contrast‐enhanced double inversion recovery (DIR) MRI sequence, which achieves robust mLV assessment in both preclinical animal models and clinical settings. Our findings reveal that brain metastatic lesions of lung adenocarcinoma (LUAD) selectively disrupt mLVs surrounding the superior sagittal sinus (SSS) in mice and humans, with DCE MRI revealing impaired cerebrospinal fluid drainage to level IIA cervical lymph nodes. Functionally, mLV ablation compromised intrathecal chemotherapy efficacy in mice, establishing the therapeutic relevance of lymphatic integrity. Clinically, patients with disease progression exhibited greater mLV structural disruption and drainage impairment, correlating with lymphatic dysfunction with metastatic advancement. Interestingly, although anti‐angiogenic agents induced mLV regression in healthy mice [19], they did not further compromise already disrupted mLVs in mice with LUAD brain metastases. These findings establish a clinic method for analyzing mLV structure and drainage, and position mLVs as prognostic indicators and potential therapeutic targets for brain metastasis management.

## 2 Results

### 2.1 DIR MRI Effectively Detected mLVs Structural Changes

Gadobutrol‐enhanced T2‐FLAIR and T1‐weighted BB MRI have demonstrated efficacy in visualizing mLVs and their CSF drainage pathways in humans and nonhuman primates [5, 16, 20]. However, these sequences show limited sensitivity for quantitative mLV volume assessment. In comparison, DIR suppresses both CSF and white matter signals to enhance the detection of subtle lesions near the cortical surface [21], which offers a promising alternative for improved quantitative assessment. We therefore optimized the DIR sequence and systematically compared its performance with FLAIR and BB sequences.

**FIGURE 1:**
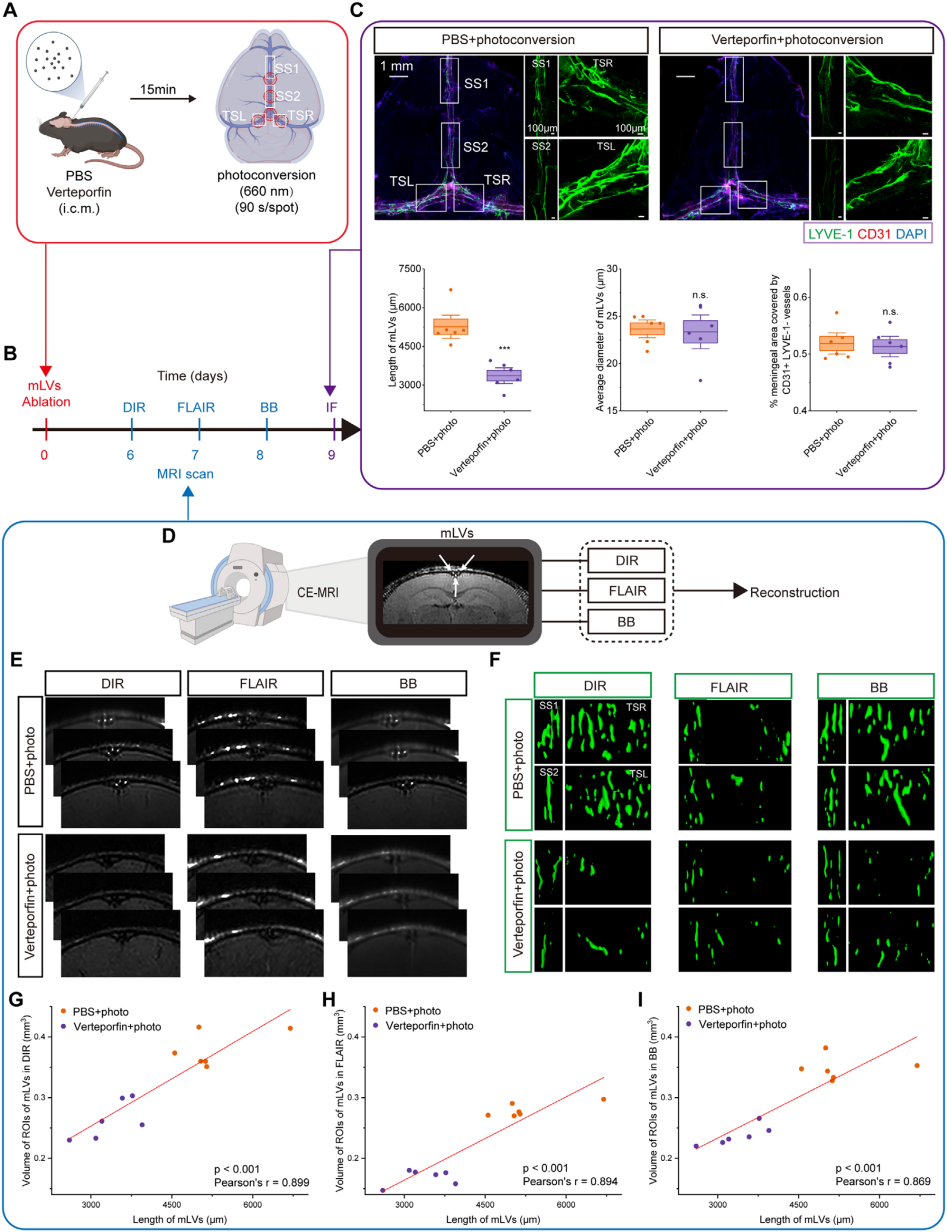
DIR MRI provides greater visualization of mLVs compared to FLAIR and BB imaging. (A) 7‐week‐old C57BL/6J mice underwent meningeal lymphatic ablation or control procedures. (B) Mice were scanned with contrast‐enhanced DIR MRI on day 6 post‐ablation, contrast‐enhanced FLAIR MRI on day 7, contrast‐enhanced BB MRI on day 8, and the meninges were harvested for analysis on day 9. (C) Representative whole‐mount meningeal images stained for LYVE‐1/CD31/DAPI. Quantification of length and average diameter of mLVs within ROIs; and area fraction (%) occupied by CD31+LYVE‐1– blood vessels in meninges. n = 6 mice/group. Data are mean ± s.e.m. ^***^ *p* < 0.001, n.s. not significant (two‐tailed unpaired Student's t‐test). (D) Mice underwent contrast‐enhanced DIR, FLAIR, and BB MRI scans for quantitative assessment of meningeal lymphatic volume. Arrows indicate mLVs. (E) Representative DIR, FLAIR, and BB images of mLVs‐SSS following gadobutrol administration in the ablation and control groups. (F) Representative 3D reconstructions of mLVs‐SSS/TS based on images of DIR, FLAIR, and BB MRI. ROIs correspond to Figure 1C. (G‐I) Correlations between immunofluorescence‐quantified mLV lengths and MRI‐quantified mLV volumes in matched regions based on DIR (G), FLAIR (H), and BB (I) (Pearson correlation analysis). n = 12/sequence.

Imaging accuracy was validated using targeted mLV ablation in mice through CSF injection of the photosensitizing agent Verteporfin, which induces mLV‐specific regression upon photoconversion (Figure 1A–C) [18]. Serial MRIs performed 6–8 days following post‐ablation revealed mLV structural alterations (Figure 1D–F). Correlation analysis between MRI‐quantified mLV volumes and immunofluorescence‐quantified mLV lengths in matched regions demonstrated significant positive correlations across all sequences (Figure 1G–I). DIR MRI sequence exhibited the strongest correlation with immunofluorescence measurements (r = 0.899, *p* < 0.001) (Figure 1G), demonstrating that DIR offers high concordance with histological findings, comparable to FLAIR (r = 0.894) and BB (r = 0.869) sequences.

### 2.2 LUAD Brain Metastases Disrupted mLVs

**FIGURE 2:**
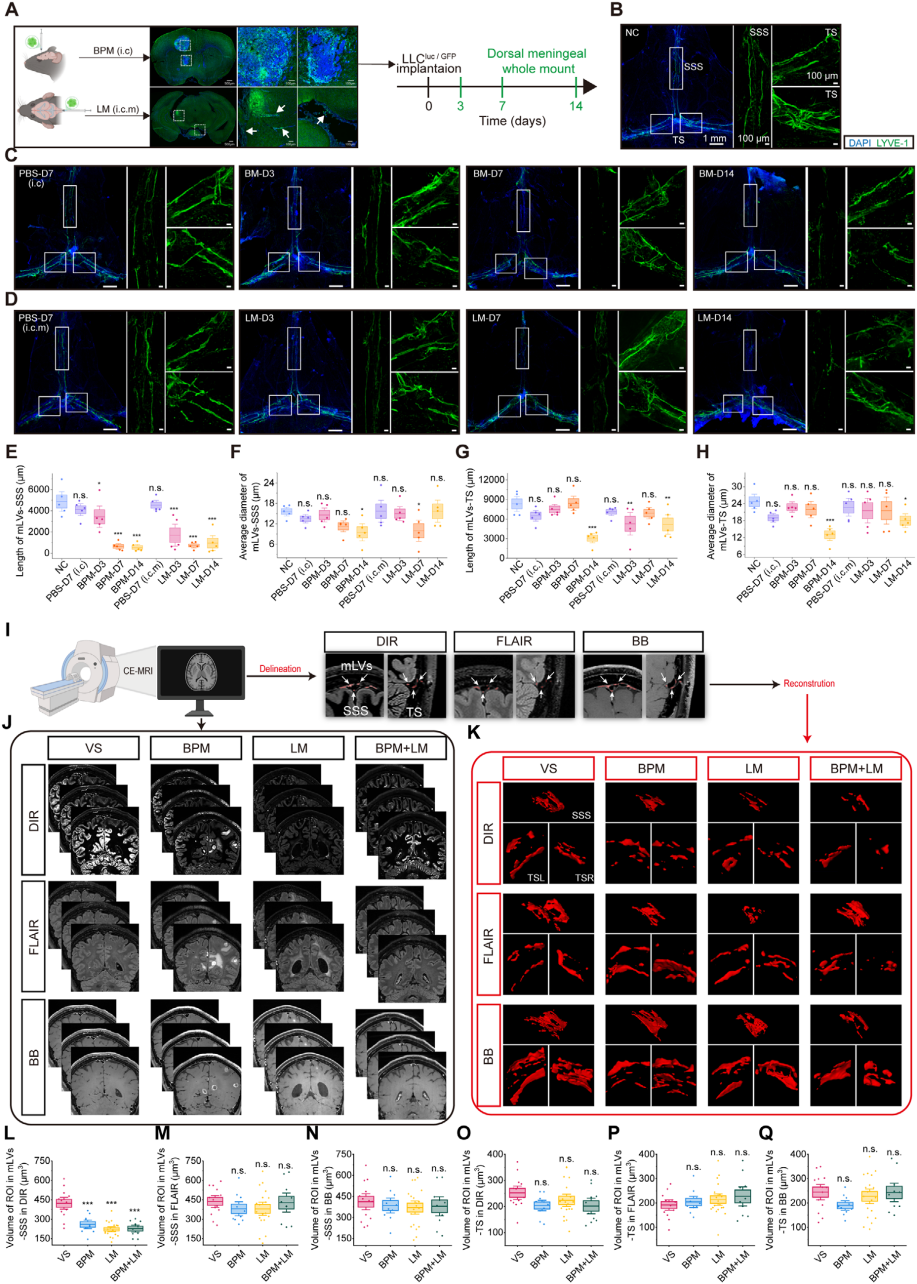
Lung adenocarcinoma brain metastases selectively disrupt mLVs‐SSS while preserving mLVs‐TS. (A) Experimental timeline for brain metastasis modeling and mLV analysis in 7‐week‐old C57BL/6J mice. (B) Representative whole‐mount meningeal images stained for LYVE‐1/DAPI in WT mice (negative controls, NC). (C) Representative LYVE‐1/DAPI‐stained meningeal whole‐mounts 3, 7, or 14 days after intraparenchymal injection of LLC ^GFP + Luc^ cells (brain parenchymal metastasis group, BPM), or 7 days post‐PBS injection. (D) Representative LYVE‐1/DAPI‐stained meningeal whole‐mounts 3, 7, or 14 days post‐intracisternal magna (i.c.m.) injection of LLC ^GFP + Luc^ cells (LM group), or 7 days post‐i.c.m. PBS injection. (E‐H) Quantification of length and average diameter of LYVE‐1+ mLVs‐SSS/TS. n = 5 mice/group. Data are mean ± s.e.m. ^*^ *p* < 0.05; ^**^ *p* < 0.01; ^***^ *p* < 0.001, n.s. not significant (one‐way ANOVA with Fisher's LSD post‐hoc test). (I) All participants underwent contrast‐enhanced DIR, T2‐FLAIR, and BB MRI for quantitative mLV volumetry. Irregular red circles denote mLVs. (J) Representative DIR, T2‐FLAIR, BB images of mLVs post‐gadobutrol in VS, BPM, LM, and combined BPM+LM patients. (K) Representative 3D reconstructions of mLVs‐ SSS/right TS/left TS within ROIs based on DIR, FLAIR, and BB images. (L‐Q) Quantification of mLVs‐ SSS/TS volume in VS (n = 13), BPM (n = 10), LM (n = 19), and BPM+LM (n = 10) patients based on DIR (L‐M), FLAIR (N‐O), and BB (P‐Q) sequences. Data are mean ± s.e.m. ^***^ *p* < 0.001, n.s. not significant (one‐way ANOVA with Fisher's LSD post‐hoc test).

To determine whether brain metastases affect mLV integrity, we established mouse models using Lewis lung carcinoma (LLC)‐GFP‐Luc cell inoculation. Cells were injected into either brain parenchyma (brain parenchyma metastasis, BPM) or leptomeninges (leptomeningeal metastasis, LM) (Figure 2A). Immunofluorescence staining of mLVs revealed progressive fragmentation around the SSS over time: shortened length with preserved diameter at day 3 post‐inoculation, further length reduction with decreased diameter at day 7, and complete structural disintegration by day 14 (Figure 2B–H). Notably, mLVs‐SSS exhibited greater susceptibility to disruption than transverse sinus‐adjacent mLVs (mLVs‐TS) (Figure 2B–H).

Clinical validation was performed with 13 vestibular schwannoma (VS) controls and 39 LUAD brain metastases patients, which included 10 BPM, 19 LM, and 10 combined BPM+LM patients (CONSORT diagram: Figure S1). All participants underwent post‐contrast MRI, including 3D DIR, 3D T2 FLAIR, and 3D T1 BB imaging (Figure 2I). Baseline characteristics showed no significant differences in blood pressure or heart rate between VS controls and metastasis groups (Table S1; Figure S2). Quantitative analysis using our newly developed DIR sequence revealed significant mLVs‐SSS volume reductions in brain metastasis patients compared to controls (*p* < 0.001), accompanied by structural disruption (Figure 2J–L). mLVs‐TS remained unchanged (Figure 2K,O). Notably, neither FLAIR nor BB sequences detected these differences (Figure 2K–Q), further confirming DIR's superior sensitivity for capturing LUAD brain metastases‐induced mLV destruction.

### 2.3 LUAD Brain Metastases Impaired mLV Drainage Function

**FIGURE 3:**
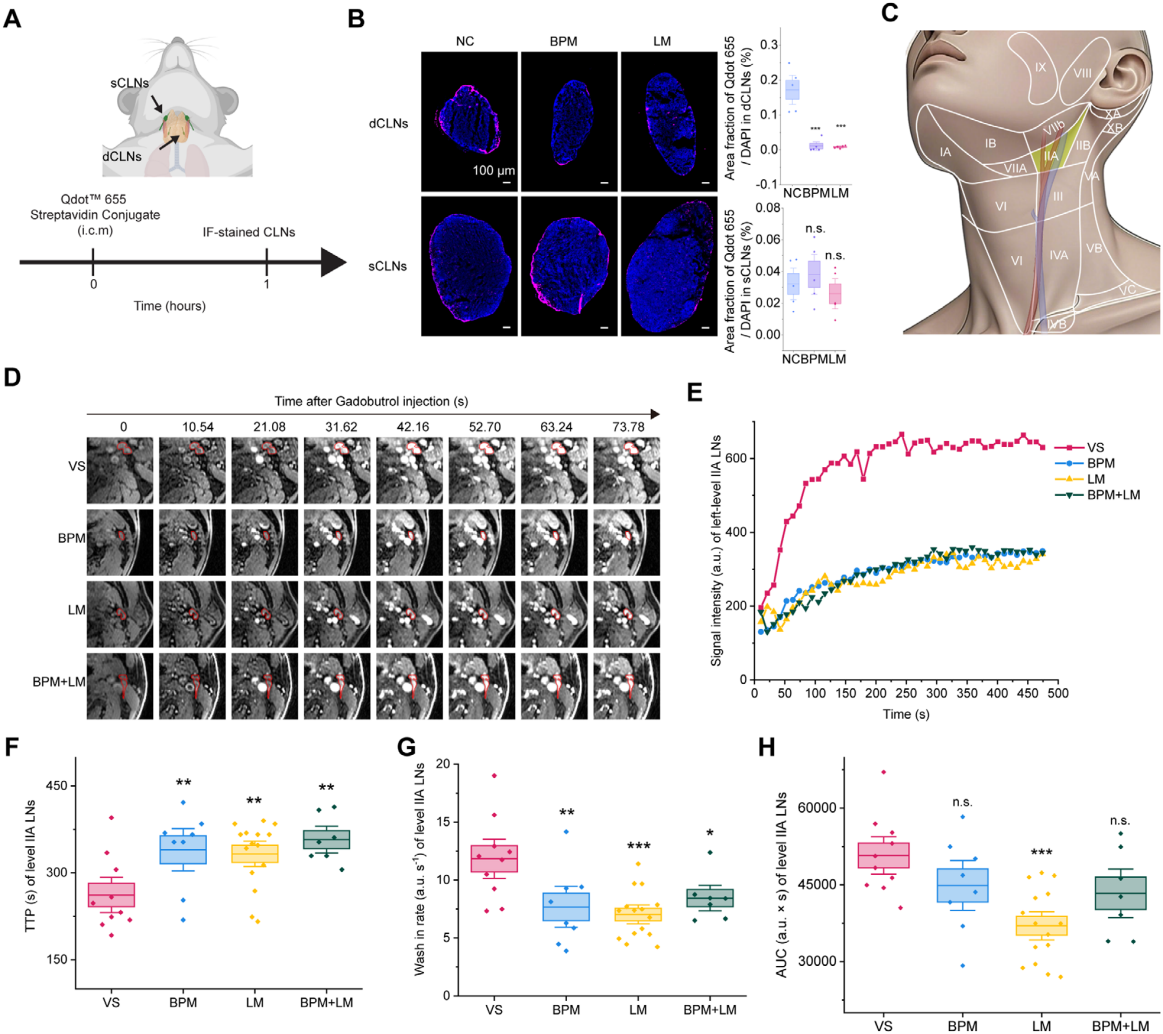
Lung adenocarcinoma brain metastases impair level IIA CLN drainage function. (A) Schematic of superficial cervical lymph node (sCLNs) and dCLNs collection 1‐h post‐i.c.m. Qdot655 injection in NC, BPM, and LM mice. (B) Left: Representative dCLN and sCLN sections showing DAPI and Qdot655 staining in NC, BPM, and LM groups. Right: Quantification of Qdot655‐positive area fraction in dCLNs and sCLNs (n = 5 mice). Data are mean ± s.e.m. ^***^ *p* < 0.001, n.s. not significant (one‐way ANOVA with Fisher's LSD). (C) Schematic of the ten levels of CLNs in humans. (D) Representative DCE‐MRI scans of left level IIA CLNs in VS, BPM, LM, and BPM+LM groups. Irregular red circles denote level IIA CLNs. (E) Representative time‐intensity curves (TICs) for left level IIA CLNs from DCE‐MRI in VS, BPM, LM, and BPM+LM groups (a.u. = arbitrary units). (F‐H) Comparison of mean TTP (F), wash‐in rate (G), and AUC (H) for bilateral level IIA CLNs in VS (n = 10), BPM (n = 8), LM (n = 15), and BPM+LM (n = 7) groups. Data are mean ± s.e.m. ^*^ *p* < 0.05; ^**^ *p* < 0.01; ^***^ *p* < 0.001, n.s. not significant (one‐way ANOVA with Fisher's LSD).

Since dorsal mLVs constitute the primary CSF drainage route to dCLNs [22], we assessed functional drainage using fluorescent tracer injection. Qdot 655 was injected into the mouse CSF, and tracer accumulation in dCLNs served as an indirect measure of mLV drainage function (Figure 3A). Both BPM and LM mouse models showed significantly reduced Qdot 655 signal in dCLNs, but not superficial CLNs (Figure 3B), which aligns with prior studies that mouse CSF primarily drains to dCLNs rather than superficial CLNs [4]. These findings demonstrate metastasis‐induced mLV drainage impairment.

We then analyzed mLV drainage in LUAD brain metastases patients using the established DCE‐MRI [13, 14, 15]. Analysis across 10 human CLN levels (Figure 3C) in VS controls and LUAD brain metastasis patients (including BPM, LM, and BPM+LM) demonstrated significantly prolonged time to peak (TTP) (Figure 3D–F), reduced wash‐in rate (Figure 3G), and decreased area under the curve (AUC) (Figure 3H) specifically in level IIA CLNs of metastasis patients compared to controls. Other lymph node levels (IA, III, IV, VA, VB, VIIA, VIII, XA, XB) remained unaffected (Figure S3), suggesting level IIA‐specific dysfunction.

**FIGURE 4:**
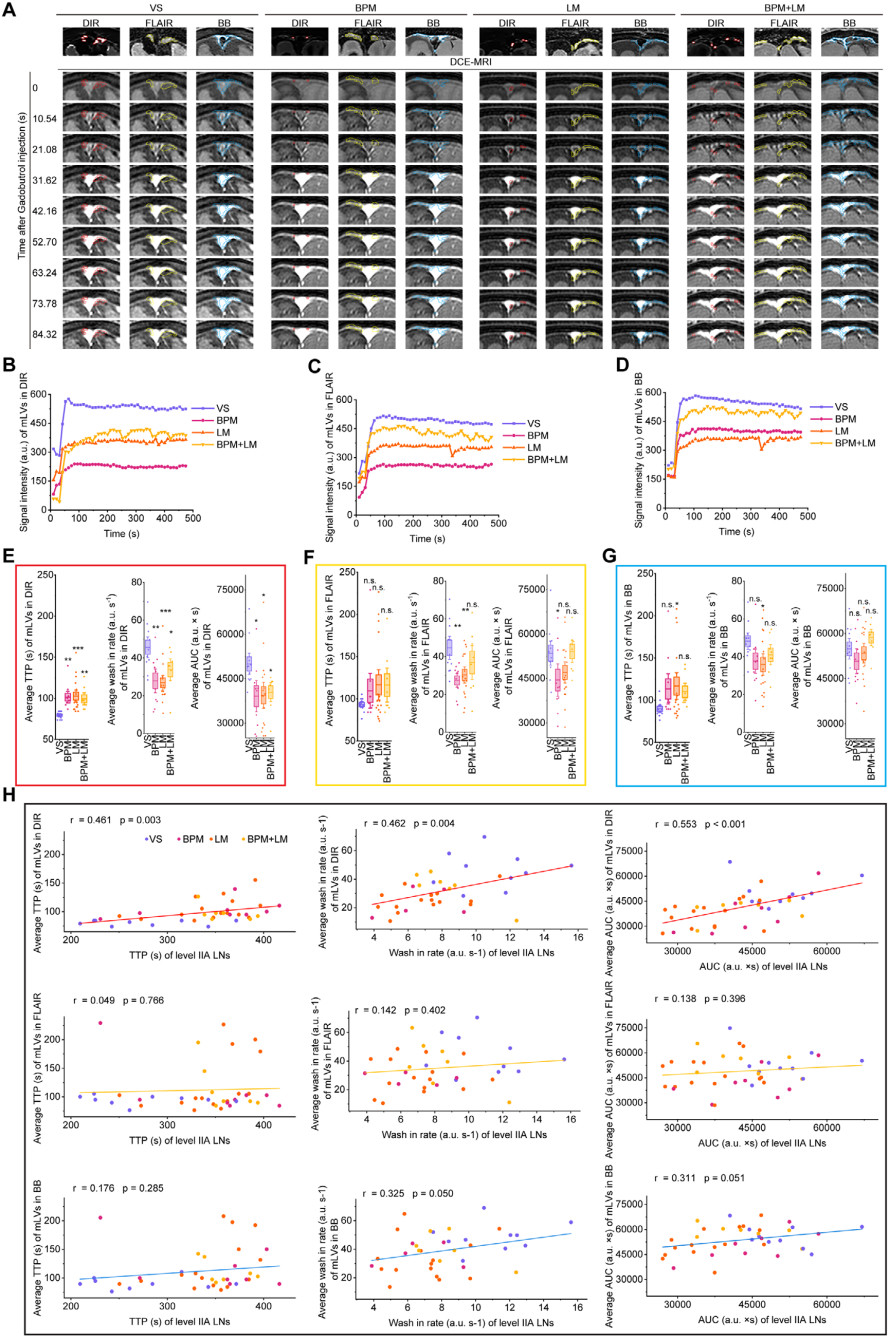
DCE‐MRI quantifies mLV drainage from SSS to level IIA CLN. (A) Representative DCE‐MRI scans of mLVs‐SSS. Irregular circles denote mLVs‐SSS localized via DIR (red), FLAIR (yellow), and BB (blue) co‐registration. (B‐D) Representative TICs for mLVs‐SSS localized via DIR (B), FLAIR (C), and BB (D) co‐registration (a.u. = arbitrary units). (E‐G) Comparison of average TTP, wash‐in rate, and AUC for mLVs‐SSS across groups: VS (n = 11), BPM (n = 10), LM (n = 17), BPM+LM (n = 8). Data are mean ± s.e.m. ^*^ *p* < 0.05; ^**^ *p* < 0.01; ^***^ *p* < 0.001, n.s. not significant (one‐way ANOVA with Fisher's LSD). (H) Correlations between level IIA CLNs parameters and co‐registered mLV‐SSS parameters (DIR/FLAIR/BB) (Pearson correlation analysis).

To investigate whether level IIA impairment originated from upstream mLV disruption, we co‐registered DIR, FLAIR, and BB MRI with DCE‐MRI to identify mLVs‐SSS (Figure 4A) and analyzed their contrast kinetics (Figure 4B–D). Metastasis patients exhibited prolonged TTP, diminished wash‐in rate, and reduced AUC in mLVs‐SSS compared to controls (Figure 4E–G), with DIR‐DCE co‐registration showing the most pronounced changes. Additionally, DIR‐DCE‐derived SSS mLV measurements correlated with level IIA CLN values (TTP: r = 0.441, *p* < 0.001; wash‐in rate: r = 0.420, *p* < 0.001; AUC: r = 0.527, *p* < 0.001) (Figure 4H). By contrast, FLAIR‐DCE showed no correlations, and BB‐DCE demonstrated only partial associations (wash‐in rate and AUC only). These findings indicate that LUAD brain metastases disrupt mLVs‐SSS drainage, with level IIA CLNs representing their primary downstream drainage target.

### 2.4 Anti‐Angiogenic Agents Did Not Further Compromise Metastasis‐Disrupted mLVs

**FIGURE 5:**
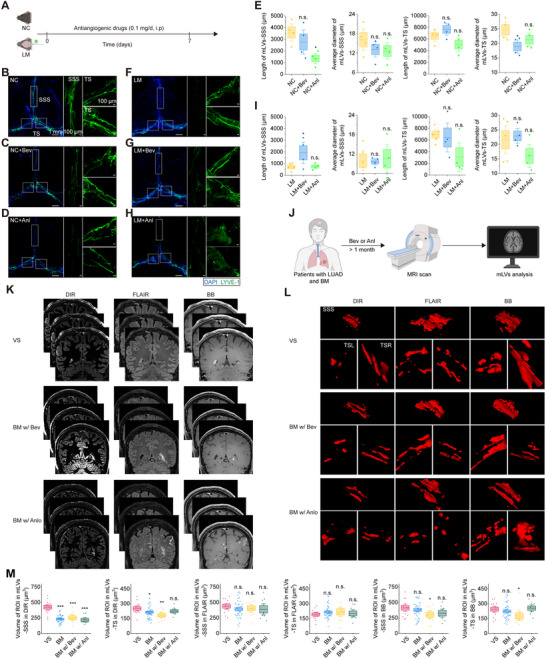
Anti‐angiogenic therapy does not exacerbate metastasis‐induced mLV disruption in lung adenocarcinoma brain metastases. (A) Experimental treatment schematic. (B‐D) Representative LYVE‐1/DAPI‐stained meninges from C57BL/6J mice treated with vehicle (B), bevacizumab (C), or anlotinib (D). (E) Quantification of LYVE‐1+ mLVs‐SSS/TS length/diameter in C57BL/6J mice treatment groups (n = 4 mice). Data are mean ± s.e.m. ^*^ *p* < 0.05, n.s. not significant (one‐way ANOVA with Fisher's LSD). (F‐H) Representative meningeal images from tumor‐bearing mice (LM) treated with vehicle (F), bevacizumab (G), or anlotinib (H). (I) Quantification of LYVE‐1+ mLVs‐SSS/TS length/diameter in tumor‐bearing mice treatment groups (n = 4 mice). Data are mean ± s.e.m. ^*^ *p* < 0.05, n.s. not significant (one‐way ANOVA with Fisher's LSD). (J) High‐resolution structural MRI for mLV volumetry in LUAD brain metastases patients receiving ≥1 month of bevacizumab/anlotinib therapy. (K) Representative DIR/FLAIR/BB images of mLVs in VS and bevacizumab/anlotinib‐treated/untreated LUAD brain metastases patients. (L) Representative 3D mLV‐SSS/TS reconstructions within ROIs. (M) Quantification of mLV volume in VS (n = 13), untreated LUAD brain metastases (n = 39), bevacizumab‐treated (n = 10), and anlotinib‐treated (n = 8) patients. Data are mean ± s.e.m. ^*^ *p* < 0.05, ^**^ *p* < 0.01; ^***^ *p* < 0.001, n.s. not significant (one‐way ANOVA with Fisher's LSD).

Given that anti‐angiogenic drugs can cause mLV regression in healthy mice [19], we investigated whether bevacizumab or anlotinib would further compromise mLVs‐SSS, which were already disrupted by LUAD brain metastases (Figure 5A). In healthy mice, seven‐day bevacizumab treatment (0.1 mg/kg/day intraperitoneally) did not significantly the length or diameter of mLVs‐SSS, though the diameter of mLVs‐TS decreased (Figure 5B, C, and E). Anlotinib treatment (0.1 mg/kg/day intraperitoneally) significantly reduced mLVs‐SSS length while leaving mLVs‐TS unaffected (Figure 5B, D, and E).

In LM mouse models where mLVs‐SSS were disrupted by tumor (Figure 2D–H), bevacizumab treatment partially restored tumor‐induced mLV length reduction without affecting mLV diameter (Figure 5F, G, and I), while anlotinib treatment showed no effect (Figure 5F, H, and I). Because neither drug exhibited cytotoxicity against LLC‐GFP‐Luc cells in vitro (Figure S4A,B) or inhibited tumor growth in vivo (Figure S4C–E), the observed mLV protection by bevacizumab appears to be a direct action on lymphatic vessels rather than a secondary consequence of tumor suppression.

Clinically, DIR MRI‐quantified mLV volumes revealed no significant differences between anti‐angiogenic‐treated and untreated LUAD brain metastasis patients (Figure 5J–M). These results demonstrate that bevacizumab and anlotinib do not induce additional regression of metastasis‐disrupted mLVs under standard therapeutic conditions.

### 2.5 Meningeal Lymphatic Integrity Correlated with Disease Progression in LUAD Brain Metastasis

**FIGURE 6:**
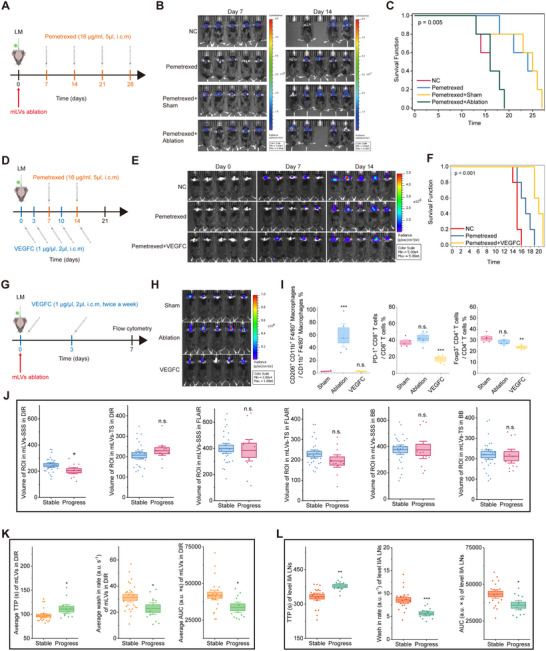
mLV impairment predicts short‐term disease progression in lung adenocarcinoma brain metastases patients. (A) Schematic of treatment plans: LLC ^GFP + Luc^ cells injected i.c.m. in C57BL/6J mice followed by mLVs ablation. Intrathecal Pemetrexed (16 µg/ml, 5 µl weekly) was initiated at day 7. (B) Tumor growth kinetics (n = 5/group) in plan (A). (C) Kaplan‐Meier survival curves (n = 5/group) in plan (A). (D) Schematic of treatment plans: C57BL/6J mice that had been injected i.c.m. with LLC ^GFP + Luc^ cells followed by intrathecal VEGFC (1 µg/µl, 5 µl, day 0, 3, 7, 10, and 14) and Pemetrexed (16 µg/ml, 5 µl weekly). (E) Tumor growth kinetics (n = 5/group) in plan (D). (F) Kaplan‐Meier survival curves (n = 5/group) in plan (D). (G) Experimental design: LLC ^GFP + Luc^ cells injected i.c.m. in C57BL/6J mice followed by mLVs ablation and intrathecal VEGFC (1 µg/µl, 5 µl, day 0 and 3). Flow cytometry was performed at day 7. (H) Tumor growth kinetics (n = 5/group) in experiment (G). (I) Quantitation of CD206^+^ macrophages as percentages of overall CD11b^+^ F4/80^+^ macrophages, PD1^+^ CD8^+^ T cells as percentages of overall CD8^+^ T cells, and Foxp3^+^ CD4^+^ T cells as percentages of overall CD4^+^ T cells in tumors. n = 5/group. Data are presented as means ± s.e.m. ^**^ *p* < 0.01, ^***^ *p* < 0.001, n.s. not significant (one‐way ANOVA with Fisher's LSD). (J) Comparison of DIR/FLAIR/BB‐derived mLVs‐SSS/TS volume in progressive disease patients with LUAD brain metastases, n (stable) = 25, n (progress) = 11. Data are mean ± s.e.m. ^*^ *p* < 0.05, n.s. not significant (two‐tailed unpaired Student's t‐test). (K) Comparison of TTP, wash‐in rate, and AUC for DIR derived mLVs‐SSS in progressive disease patients with LUAD brain metastases. n (stable) = 24, n (progress) = 11. Data are mean ± s.e.m. ^*^ *p* < 0.05 (two‐tailed unpaired Student's t‐test). (L) Comparison of TTP, wash‐in rate, and AUC for level IIA CLNs in progressive disease patients. Data are mean ± s.e.m. ^*^ *p* < 0.05; ^**^ *p* < 0.01; ^***^ *p* < 0.001, n (stable) = 19, n (progress) = 11. (two‐tailed unpaired t‐test).

To investigate whether mLV disruption affects therapeutic response, we examined intrathecal pemetrexed efficacy following either mLV ablation (Figure 6A) or enhancement (Figure 6D) in a mouse model of LM. As expected, intrathecal pemetrexed alone inhibited tumor growth and prolonged survival. However, pre‐treatment mLV ablation via verteporfin significantly attenuated these therapeutic benefits (Figure 6B,C). Conversely, pre‐treatment mLV enhancement via VEGFC improved these therapeutic effects (Figure 6E,F), demonstrating that mLV integrity is critical for treatment efficacy. Mechanistically, flow cytometry analysis revealed distinct immunological shifts driven by mLV modulation. mLV ablation led to a significant increase in the proportion of CD206^+^ tumor‐associated macrophages (TAMs) (Figure 6G–I and Figure S6A,B), suggesting a shift toward an immunosuppressive M2‐like phenotype that may hinder anti‐tumor immunity. In contrast, mLV enhancement via VEGFC resulted in a significant reduction in the proportions of PD‐1^+^CD8^+^ T cells and Tregs (Figure 6G–I and Figure S6A,B), indicating an alleviation of the immunosuppressive tumor microenvironment.

Building on these preclinical findings, we next examined the relationship between disease progression and mLV integrity in human patients. Disease status was classified as stable disease or progressive disease based on RANO criteria, assessed three months post‐MRI in LUAD brain metastasis patients. Notably, progressive disease cases exhibited significantly reduced DIR MRI‐quantified mLVs‐SSS volumes compared to stable disease cases. No such differences were observed in TS regions or with BB/FLAIR sequences (Figure 6J). Functional imaging analysis using DIR‐DCE co‐registration further demonstrated prolonged TTP, diminished wash‐in rate, and reduced AUC in mLVs‐SSS in progressive disease patients (Figure 6K), which were absent in BB‐DCE or FLAIR‐DCE analyses. Similarly, level IIA CLNs were more severely impaired in progressive disease patients compared to stable disease patients, with prolonged TTP, decreased wash‐in, and reduced AUC (Figure 6L). Importantly, binary logistic regression analysis identified the average wash‐in rate of level IIA CLNs as an independent predictor of short‐term disease progression (OR: 0.379, 95% CI: 0.161–0.887, p = 0.025; Table S2), indicating that higher wash‐in rates are associated with a reduced risk of short‐term progression.

Collectively, these results suggest that structural and functional mLV impairment, as detected by our newly developed DIR MRI sequence, significantly correlates with disease progression in LUAD brain metastasis patients, establishing mLV integrity as a potential predictor of clinical outcomes.

## 3 Discussion

Clinical evidence for mLV alterations in brain metastases has remained limited despite prior investigations establishing that brain tumors affect mLV structure and drainage [7, 9, 23]. Our study addresses this gap by developing optimized MRI methods that enable quantitative assessment of mLV volume and contrast uptake dynamics in both preclinical animal models and clinical settings. DIR sequence provides improved mLV visualization, where the mLVs shown in DIR are clearer due to enhanced suppression of CSF and vascular signals compared to conventional FLAIR and BB imaging. Validation in mouse mLV photo‐ablation models confirmed the reliability of DIR in detecting mLV structural changes. Subsequent clinical studies further demonstrated DIR's enhanced sensitivity for detecting mLV volume differences across patient subgroups. Although current resolution constraints (0.04 × 0.04 mm voxels in mice and 0.6‐0.8 mm voxels in humans) render DIR measurements approximate values rather than absolute values, these methods provide clinically applicable quantitative assessment of lymphatic integrity with sufficient sensitivity to detect disease‐related alterations.

The selective SSS disruption with TS preservation we observed in LUAD brain metastases contrasts with lymphangiogenesis reported in glioma and melanoma models, which demonstrate increased vessel diameter and sprouting around the TS [7, 23]. This difference likely reflects distinct pathological mechanisms between metastatic and primary tumors, as well as regional differences in lymphatic architecture and susceptibility to tumor‐induced damage. Mechanistically, mLV destruction may arise from a hostile metastatic microenvironment enriched in pro‐inflammatory cytokines and oxidative stress, which are known to destabilize lymphatic endothelial identity and integrity [24, 25]. In addition, tumor‐associated elevation of intracranial pressure may mechanically compromise mLVs‐SSS, rendering them particularly vulnerable to collapse [26]. Although cranial manipulation can induce regional lymphangiogenesis [27], our surgical controls (Figure S5) indicate that the selective mLVs‐SSS disruption observed here reflects a pathological consequence of metastasis rather than a procedural artifact.

Human CLNs are classified into 10 levels via computed tomography (CT) imaging [28]. DCE‐MRI analysis across all cervical lymph node levels (IA through XB) identified level IIA CLNs as primary drainage sites for mLVs‐SSS. Level IIA's anatomical location in the upper jugular region, anterior to the internal jugular vein and nearest the jugular foramen, aligns with drainage pathways along venous sinuses. Drainage parameters in level IIA most consistently reflected those of dorsal mLVs (Figure 4H), establishing this anatomical correlation.

Given that bevacizumab and anlotinib are commonly used anti‐angiogenic drugs for treating LUAD brain metastases [29, 30], we investigated their potential effects on mLV integrity. At concentrations non‐cytotoxic to tumor cells (Figure S4), both drugs moderately disrupted mLVs structure in healthy mice. However, in mouse models where mLVs were already compromised by brain metastases, neither bevacizumab nor anlotinib caused further mLV disruption. Similarly, in LUAD brain metastasis patients, these drugs did not induce additional mLV damage beyond tumor‐induced changes. These findings suggest that anti‐angiogenic therapy does not exacerbate metastasis‐induced lymphatic dysfunction.

Functional studies demonstrated that mLV ablation compromised intrathecal pemetrexed efficacy in a mouse LM model, which was associated with the establishment of an immunosuppressive microenvironment characterized by macrophage polarization. Clinically, patients with disease progression within three months exhibited significantly greater disruption in mLVs‐SSS and level IIA CLNs compared to stable disease patients. Although limited by sample size and the absence of external validation, our findings indicated that mLV dysfunction is an independent risk factor in the current cohort, warranting validation in larger, multi‐center studies.

## 4 Conclusion

We established optimized MRI methods for quantitative mLV assessment and revealed selective disruption of mLVs‐SSS in LUAD brain metastases. We also identified parallel impairment in level IIA CLNs that serve as primary downstream drainage sites. The correlation between lymphatic impairment and disease progression identifies mLVs as both prognostic biomarkers and potential therapeutic targets.

## 5 Materials and Methods

### 5.1 Mice

Wild‐type (WT) mice (C57BL/6J) were purchased from the Chinese Institute for Brain Research (CIBR), Beijing. Mice were housed in individual cages (3 to 5 animals per cage) in a pathogen‐free animal facility under a 12–12 h light‐dark cycle and a temperature range of 20–26°C with a humidity of 40–70%. Seven‐week‐old male mice were randomly assigned for use in this study. All experiments were conducted in accordance with the procedures approved by the Animal Care and Use Committee of CIBR, including committee‐approved humane endpoints (moribund state or ≥20% reduction from baseline body weight), none of which were exceeded during the study.

### 5.2 mLV ablation

Mice were anesthetized using 3% isoflurane for induction and maintained with 1.5% isoflurane. Animals were secured in a stereotaxic frame, the head and neck skin was shaved, and the skin at the dorsal craniocervical junction was disinfected with 0.5% iodophor. A 5‐mm midline longitudinal skin incision was made, neck muscles were separated, and the dura overlying the cisterna magna was exposed. A 10‐µL Hamilton microsyringe was used to inject 5 µL of 3 mg/mL verteporfin (V129759; Aladdin) into the cisterna magna. After 15 min, a small scalp incision was made to expose the skull. Non‐thermal red light (660 nm wavelength; MRL‐III‐660L‐100 mW laser, CNI) was applied for photoconversion at five locations on the intact skull (two over the SSS, one at the confluence of sinuses, and one over each TS). Each site was irradiated at 600 mW/cm^2^ for 90 s. Control group mice were injected with an equivalent volume of phosphate‐buffered saline (PBS) and underwent identical photoconversion. The scalp was sutured, and mice received subcutaneous meloxicam (1 mg/kg) for analgesia before recovering on a heating pad until fully ambulatory.

### 5.3 Cells

LLC cells, derived from a lung tumor in a C57BL mouse implanted with primary Lewis lung carcinoma, were acquired from the National Infrastructure of Cell Line Resource (NICR). Cells were transduced with a lentiviral vector encoding CMV‐driven GFP‐Luc to generate LLC‐GFP‐Luc cells. Cells were cultured in Dulbecco's Modified Eagle Medium (DMEM) supplemented with 10% fetal bovine serum (FBS) and 1% penicillin/streptomycin at 37°C in 5% CO_2_, and passaged every 3–4 days. All experiments used cells within 25 passages.

### 5.4 Experimental Intracranial Xenograft Model of LUAD BPM

Mice were anesthetized as described, shaved, and the calvarial scalp was disinfected with 0.5% iodophor. A 5‐mm midline longitudinal incision was made, and the underlying periosteum was removed. We drilled a 0.5‐mm diameter burr hole 1.5 mm right lateral and 1.5 mm posterior to bregma using a dental drill. A 10‐µL Hamilton microsyringe injected 5 µL containing 2.5 × 10^5^ LLC‐GFP‐Luc cells into the brain parenchyma (2‐mm depth) over 2 min. Post‐injection, the scalp was sutured and disinfected with 0.5% iodophor. Mice received subcutaneous meloxicam (1 mg/kg) for postoperative analgesia and recovered on a warming pad.

### 5.5 Experimental Intracranial Xenograft Model of LUAD LM

Using the above protocol, we injected 5 µL containing 5 × 10^4^ LLC‐GFP‐Luc cells into the cisterna magna of mice. Post‐injection, the skin was sutured and disinfected with 0.5% iodophor. Mice received subcutaneous meloxicam (1 mg/kg) for analgesia and recovered on a warming pad.

### 5.6 Craniotomy

After anesthesia, we created a 2‐mm diameter cranial burr hole over the SSS or parasagittal region using a dental drill. The cranial scalp was sutured and disinfected with 0.5% iodophor. Mice received subcutaneous meloxicam (1 mg/kg) for postoperative analgesia and recovered on a warming pad.

### 5.7 MTT Assay

We collected logarithmically growing LLC‐GFP‐Luc cells, adjusted the cell suspension concentration, and plated them in 96‐well plates (180 µL/well; 5000 cells/well). After 12‐h incubation at 37°C with 5% CO_2_ to allow adhesion, we replaced the medium with fresh medium containing bevacizumab (Ankeceda; Qilu Pharmaceutical) or anlotinib dihydrochloride (HY‐19716A; MCE) at the indicated concentrations (0, 0.00016, 0.0008, 0.004, 0.02, 0.1 mg/mL). Each concentration was tested in triplicate. After 24‐h incubation, we aspirated supernatant, added 90 µL fresh medium and 10 µL MTT solution (3‐(4,5‐dimethylthiazol‐2‐yl)‐2,5‐diphenyltetrazolium bromide; M1020, Solarbio), and incubated cells for 2 h. Following supernatant removal, we added 110 µL dimethyl sulfoxide (DMSO) per well. Plates were shaken at low speed for 10 min to dissolve formazan crystals. Absorbance was measured at 490 nm using a microplate reader (Spark; TECAN). Blank wells (medium + MTT + DMSO) served for zeroing.

### 5.8 Anti‐Angiogenic Drug Treatment

We administered bevacizumab and anlotinib (0.1 mg/kg/day) via intraperitoneal (i.p.) injection to WT mice and LM‐bearing mice for 7 consecutive days.

### 5.9 Tumor Growth Monitoring

We performed bioluminescence imaging using an in vivo imaging system (IVIS Lumina III; Revvity) to monitor LLC‐GFP‐Luc tumor growth. Mice were anesthetized with 3% isoflurane (induction) and maintained at 1.5% isoflurane. We administered 200 µL of D‐luciferin (15 mg/mL; LUCK‐100, GoldBio) intraperitoneally. After 10 min, anesthetized mice were imaged using IVIS. Region‐of‐interest (ROI) signal intensity was quantified using Living Image software (v4.7.3; PerkinElmer).

### 5.10 Survival Monitoring

We measured body weight daily. Humane endpoints were defined as ≥20% body weight loss from baseline or spontaneous death.

### 5.11 Dura Mater Collection

We anesthetized mice with tribromoethanol (500 mg/kg, i.p.) and perfused them sequentially through the left ventricle with PBS and 4% paraformaldehyde (PFA). Under microscopic guidance, we dissected intact dorsal dura mater. Meninges were fixed in 4% PFA for 12 h, then stored in PBS with 0.02% sodium azide at 4°C.

### 5.12 CLN Drainage and Collection

Mice anesthetized with 3% isoflurane (induction) and maintained at 1.5% isoflurane were secured in a stereotaxic frame. After shaving and disinfecting the region at the dorsal craniocervical junction with 0.5% iodophor, we made a 5‐mm midline incision, separated neck muscles, and exposed the cisterna magna. Using a 10‐µL Hamilton microsyringe, we injected 5 µL Qdot 655 Streptavidin Conjugate (Q10123MP; Invitrogen) into the cisterna magna. The incision was sutured and sterilized. Mice received subcutaneous meloxicam (1 mg/kg) for analgesia and recovered on a warming pad. 1‐h post‐injection, we anesthetized mice with tribromoethanol (500 mg/kg, i.p.) and performed cardiac perfusion with PBS followed by 4% PFA. We microscopically dissected superficial and dCLNs, fixed them in 4% PFA for 24 h, washed with PBS, dehydrated in 30% sucrose, and embedded in Tissue‐Plus OCT compound. Using a cryostat (CM3050 S; Leica), we sectioned CLNs at 10‐µm thickness, mounted sections on gelatin‐coated slides, and stored them at ‐20°C.

### 5.13 Meningeal and CLN Immunofluorescence Staining and Imaging

We washed tissues with PBS, blocked them for 2 h in buffer containing 5% donkey serum, 2% bovine serum albumin (BSA), 0.5% Triton X‐100, and 0.05% Tween‐20. Tissues were incubated overnight at 4°C with primary antibodies diluted in the same blocking buffer: Anti‐LYVE‐1 (1:100; ab14917, Abcam, Cambridge, UK), Anti‐CD31 (1:100; AF3628, R&D Systems, Minneapolis, MN). After four 10‐min PBS washes at room temperature (RT), we incubated tissues with secondary antibodies in PBS containing 0.5% Triton X‐100 for 2 h at RT: Donkey anti‐rabbit IgG (H+L) Alexa Fluor 594 (1:500; A21207, Invitrogen, Waltham, MA), Donkey anti‐goat IgG (H+L) Alexa Fluor 488 (1:500; #705‐545‐147, Jackson ImmunoResearch, West Grove, PA). Following three 5‐min PBS washes, we counterstained nuclei with 4',6‐diamidino‐2‐phenylindole (DAPI) (10 µg/mL) for 15 min. After final PBS washes, we mounted slides in glycerol‐based medium and stored them at ‐20°C until imaging. We acquired images using a virtual slide scanning system (VS120; Olympus, Tokyo, Japan) with a 10 × objective.

### 5.14 Flow Cytometry

Mice were euthanized by intraperitoneal injection of tribromoethanol (500 mg/kg), followed by transcardiac perfusion with PBS. Tumors were microdissected in ice‐cold PBS, minced with scissors, and transferred into pre‐warmed HBSS containing papain (20 U/mL, Worthington), DNase I (200 U/mL, Worthington), ovomucoid protease inhibitor (10 mg/ml, Worthington), and 2% FBS (Gibco) for enzymatic dissociation. The tissue was digested for 30 min, passed through a 70‐µm cell strainer, and centrifuged at 1240 G for 3 min. Cells were resuspended in 40% percoll (17089101, Cytiva) and centrifuged at 1240 G for 5 min. Viability staining was performed using Live/Dead (Invitrogen) for 30 min at 4°C. Cells were then centrifuged at 1240 G for 3 min and resuspended in anti‐CD16/32 antibody solution (1:100, eBioscience) diluted in cold PBS to block Fc receptors for 10 min at 4°C. Cells were then centrifuged at 1240 G for 3 min, and surface staining was performed by adding fluorophore‐conjugated antibodies diluted in PBS for 30 min on ice: anti‐CD45‐BV650 (1:100; 563410, BD Horizon), anti‐TCRβ‐PE (1:100; 12‐5961‐82, eBioscience), anti‐CD4‐APC (1:100; 17–004181, eBioscience), anti‐PD1‐BV510 (1:100; 135241, Biolegend), anti‐CD11b‐BV711 (1:100; 407‐0112‐82, eBioscience), anti‐F4/80‐PE/Dazzle594 (1:100; 123146, Biolegend), anti‐CD206‐PerCP‐Cy5.5 (1:100; 141716, Biolegend). Then, anti‐Foxp3‐eFluor450 (1:100; 48‐5773‐80, eBioscience) staining was performed using the transcription factor Foxp3 staining kit (Tonbo). Samples were resuspended in cold PBS and acquired and analyzed on an LSR Fortessa flow cytometer (BD Biosciences) and Flow Jo software (v.10, BD Biosciences).

### 5.15 Mouse MRI

We injected gadobutrol (Gadovist; Bayer), a gadolinium‐based contrast agent (100 µL), intravenously via the tail vein. Mice were anesthetized with 3% isoflurane (induction) and positioned in a 9.4T MRI scanner (uMR; United Imaging). During imaging, anesthesia was maintained with 1.5% isoflurane via nose cone, with body temperature regulated by a warming pad. Beginning 8 min post‐injection, we acquired the following sequences for mLV visualization:

#### 5.15.1 Double Inversion Recovery (DIR)

Repetition time (TR) = 2000 ms; echo time (TE) = 156.98 ms; inversion time (TI) = 956 ms; echoes = 1; averages = 1; scan time = 14 min 18 s; flip angle = 56°; field of view (FOV) = 19.2 × 19.2 × 17.6 mm; matrix = 480 × 480 × 220; voxel size = 0.04 × 0.04 × 0.08 mm; slices = 220.

#### 5.15.2 T2‐Fluid‐Attenuated Inversion Recovery (FLAIR)

TR = 3000 ms; TE = 136.94 ms; TI = 1150 ms; echoes = 1; averages = 1; scan time = 17 min 54 s; flip angle = 102°; FOV = 19.2 × 19.2 × 17.6 mm; matrix = 480 × 480 × 220; voxel size = 0.04 × 0.04 × 0.08 mm; slices = 220.

#### 5.15.3 T1‐Weighted Black‐Blood (BB)

TR = 800 ms; TE = 10.02 ms; echoes = 1; averages = 1; scan time = 15 min 20 s; flip angle = 89°; FOV = 19.2 × 19.2 × 17.6 mm; matrix = 480 × 480 × 220; voxel size = 0.04 × 0.04 × 0.08 mm; slices = 220.

### 5.16 MRI Processing

In Verteporfin mLV ablation experiments, we selected ROIs surrounding SSS (1–3.5 and 4.5–7 mm posterior to torcula) and TS (0.5–2.5 mm lateral to torcula) for both control and ablation groups. We segmented and calculated mLV volumes within ROIs using ITK‐SNAP software (v4.2.0; www.itksnap.org). Data acquisition/processing was performed by masked researchers.

### 5.17 Fluorescence Image Processing

We conducted quantitative image analysis using ImageJ software (v1.54p; 64‐bit Java 1.8.0_322; National Institutes of Health, USA), measuring mLV dimensions within predefined anatomical regions relative to the torcula: specifically, length and average diameter were quantified along the SSS 2–5 mm posteriorly and the TS 0.5–2.5 mm laterally. For mLVs ablation experiments, these measurements were extended to additional SSS regions (1–3.5 and 4.5–7 mm posteriorly) while maintaining TS assessment at 0.5–2.5 mm laterally. Concurrently, we quantified fluorescent tracer drainage in CLNs as area fraction.

### 5.18 Approval and Patient Informed Consent

This study was approved by the Institutional Ethics Committee of Beijing Tiantan Hospital, Capital Medical University (KY202307302). Written informed consent was obtained from all participants or their legal guardians.

### 5.19 Patient Recruitment

Patient selection and exclusion criteria are detailed in Figure S1. We recruited fourteen VS patients (benign extracranial tumors, Koos grade II‐III [31]) as controls from the Second Neuro‐Oncology Ward and seventy‐eight suspected brain metastasis patients from the Department of Oncology at Beijing Tiantan Hospital, Capital Medical University. All participants underwent physical examination, electrocardiography (ECG), and laboratory testing, including complete blood count, urinalysis, hepatic/renal function, glucose/lipid panels, and thyroid function assessments, with MRI confirming diagnoses. We excluded eleven metastasis‐suspected patients lacking LUAD brain metastases: four without intracranial abnormalities, four with breast cancer metastases, one with colon cancer metastasis, one with meningioma, and one with lung squamous cell carcinoma metastasis. The final cohort comprised 81 patients: 14 VS controls, 21 LUAD BPM patients, 28 LUAD LM patients, and 18 combined BPM+LM patients. Brain metastasis diagnosis followed established MRI criteria [32, 33], though final MRI scans were obtained from only 13 VS, 20 BPM, 22 LM, and 15 BPM+LM patients due to exclusions from blurred images, corrupted files from scanner malfunction, or patient non‐compliance during scanning. Additionally, two attending neurologists and one psychiatrist confirmed that control patients lacked comorbid neurological/psychiatric disorders.

### 5.20 MRI Scanning Protocol

We monitored vital signs (blood pressure, heart rate) pre‐ and post‐MRI. Scans used a 3 Tesla scanner (uMR‐NX890; United Imaging Healthcare) with 64‐channel head‐neck coil. Participants received intravenous gadobutrol (Gadovist; Bayer; 0.1 mmol/kg) via power injector (MEDRAD MRXperion; Bayer) at 3 mL/s.

### 5.21 MRI sequences:

#### 5.21.1 Structural mLV Assessment

##### 5.21.1.1 Double Inversion Recovery (DIR)

Coronal 3D; TR = 5500 ms, TE = 294.84 ms; FOV = 256 mm; matrix = 435 × 480; voxel = 0.8 × 0.8 × 0.8 mm^3^; 240 slices (0.8 mm); time = 7 min 48 s.

##### 5.21.1.2 T2‐Fluid‐Attenuated Inversion Recovery (FLAIR)

Coronal 3D; TR = 6000 ms, TE = 399.64 ms; FOV = 256 mm; matrix = 427 × 480; voxel = 0.8 × 0.8 × 0.8 mm^3^; 360 slices (0.8 mm); time = 5 min 12 s.

##### 5.21.1.3 T1‐Weighted Black‐Blood (BB)

Coronal 3D; TR = 800 ms, TE = 13.26 ms; FOV = 268 mm; matrix = 552 × 672; voxel = 0.6 × 0.6 × 0.6 mm^3^; 480 slices (0.6 mm); time = 5 min 4 s.

#### 5.21.2 Drainage Assessment (mLVs/CLNs)

##### 5.21.2.1 Dynamic Contrast‐Enhanced MRI (DCE‐MRI)

Coronal 2D T1‐GRE; 45 phases (10.54 s/phase); 200 slices (1.5 mm); TR = 5.1 ms, TE = 2.24 ms; FOV = 250 mm; matrix = 358 × 408; voxel = 0.92 × 0.92 × 1.5 mm^3^; time = 8 min 30 s. Contrast injection after 2 baseline phases.

Following gadolinium‐based contrast agent injection, we acquired images sequentially using: DCE‐MRI, DIR, BB and FLAIR.

### 5.22 Clinical Follow‐Up

At 3 months post‐MRI, we evaluated therapeutic responses in two distinct cohorts comprising 18 patients receiving bevacizumab or anlotinib therapy alongside 39 untreated LUAD brain metastasis patients, with all clinical responses assessed using the simplified Response Assessment in Neuro‐Oncology (RANO) criteria [34, 35] to establish short‐term disease progression status.

### 5.23 MRI Analysis

Given that anti‐angiogenic agents (bevacizumab or anlotinib) may induce reversible mLVs regression [19] and potentially confound clinical analyses, we restricted mLV assessment exclusively to structural analysis for the 18 drug‐treated patients while analyzing structural data from 13 VS, 10 BPM, 19 LM, and 10 BPM+LM patients after excluding anti‐angiogenic‐treated cases; drainage analysis incorporated 11 VS, 10 BPM, 17 LM, and 8 BPM+LM patients despite six exclusions due to blurred DCE‐MRI images, with subsequent correlation of mLV structure/drainage against short‐term progression in 10 BPM, 19 LM, and 7 BPM+LM metastasis patients after excluding three lost‐to‐follow‐up individuals.

### 5.24 Image Processing

Two blinded radiologists independently processed motion‐corrected images using ITK‐SNAP to manually segment and calculate mLV volumes within anatomically defined ROIs—specifically the SSS region spanning 7–27 mm anterior to torcula (20 mm slab) and TS region spanning 12–27 mm anterior to torcula (15 mm slab)—deriving volumes from post‐contrast DIR, FLAIR, and BB sequences based on signal intensity. Through United Imaging Workstation (uWS‐MR: R005), we assessed contrast uptake dynamics in mLVs and CLNs, measuring mLV drainage via contrast uptake in three consecutive SSS/TS‐registered slices after DCE co‐registration with structural sequences (DIR/FLAIR/BB) while identifying CLNs across levels IA, IIA, III, IV, VA, VB, VIIA, VIII, XA, XB on BB‐co‐registered DCE; quantitative uptake parameters included time‐to‐peak (TTP, contrast infusion initiation to maximum signal intensity), wash‐in rate (maximum signal‐rise slope), and area under the curve (AUC, baseline‐subtracted time‐signal integral) as detailed in Figure S2.

### 5.25 Statistics

We performed all statistical analyses using OriginPro software (v.2021, 9.8.0.200; OriginLab, Northampton, MA) and IBM SPSS Statistics (v.27.0; IBM Corp., Armonk, NY). Categorical variables (e.g., sex) were presented as frequencies (percentages), while continuous demographic data were expressed as mean ± standard deviation (SD) or mean ± standard error (SE). To evaluate the impact of lung adenocarcinoma brain metastases on mLV structure and drainage, we employed analysis of variance (ANOVA). Independent samples t‐tests and chi‐square tests assessed intergroup differences and clinical factor‐outcome associations. Linear regression with Pearson correlation analysis quantified concordance between fluorescence‐imaging‐derived and MRI‐derived mLV structure. To identify independent risk factors associated with disease progression, binary logistic regression analysis was performed. Variables yielding a *p* < 0.1 in the univariate analysis were subsequently included in a multivariate logistic regression model utilizing a forward elimination procedure. Results were reported as estimated odds ratios (OR) with corresponding 95% confidence intervals (CI). Statistical significance was defined as *p* < 0.05. All visualizations were generated in OriginPro.

## Author Contributions

**W.J**., **Y.Z**., **W.S**., and **D.L.L**. conceptualized the study. **Y.Z**., **S.C.J**., **P.H.G**., **J.J**., **Z.Z**., **S.C**., **X.H**., **B.W.L**., and **Y.L**. carried out the experiments. **X.Y.L**., **Y.C.H**., and **X.S.D**. recruited patients. **Y.Z**., **S.C.J**., **P.H.G**., and **Y.Y.Y**. analyzed data and participated in the interpretation of results. **D.L.L**. and **Y.Z**. acquired the funding for this research. **Y.Z**., **S.C.J**., and **P.H.G**. curated the data. **Y.Z**. and **W.S**. prepared the initial manuscript draft and revised it based on feedback and suggestions from all authors. All authors have read and approved the article.

## Declaration of Generative AI and AI‐assisted Technologies

During the preparation of this work, the authors used DeepSeek R1 and Grok3 to check the grammar of the manuscript. After using these tools, the authors reviewed and edited the content as needed and takes full responsibility for the content of the publication

## Conflicts of Interest

The authors declare no conflicts of interest.

## Supporting information




**Supporting File**: advs73642‐sup‐0001‐SuppMat.docx.

## Data Availability

The data that support the findings of this study are available from the corresponding author upon reasonable request.
