# Supplementary material for: Magnetic Resonance Imaging Reveals Meningeal Lymphatic Impairment in Lung Adenocarcinoma Brain Metastasis Progression
Source: Adv Sci (Weinh). 2026 Jan 15;13(16):e16988. doi: 10.1002/advs.202516988 (PMC13042558; doi:10.1002/advs.202516988)
Supplement: Supplementary file 1 — Supporting File: advs73642‐sup‐0001‐SuppMat.docx. [file ADVS-13-e16988-s001.docx]

Supplementary Materials

Magnetic Resonance Imaging Reveals Meningeal Lymphatic Impairment in Lung Adenocarcinoma Brain Metastasis Progression

Yuan Zhang, Shucheng Jin, Penghui Guo, Yuying Yin, Yichun Hua, Xiaosheng Ding, Zhe Zhang, Shuo Chen, Xu Han, Baowang Li, Yan Liu, Xiaoyan Li, Deling Li, Jing Jing, Wei Shi, Wang Jia.

Correspondence to: jwttyy@126.com

Figure. S1.


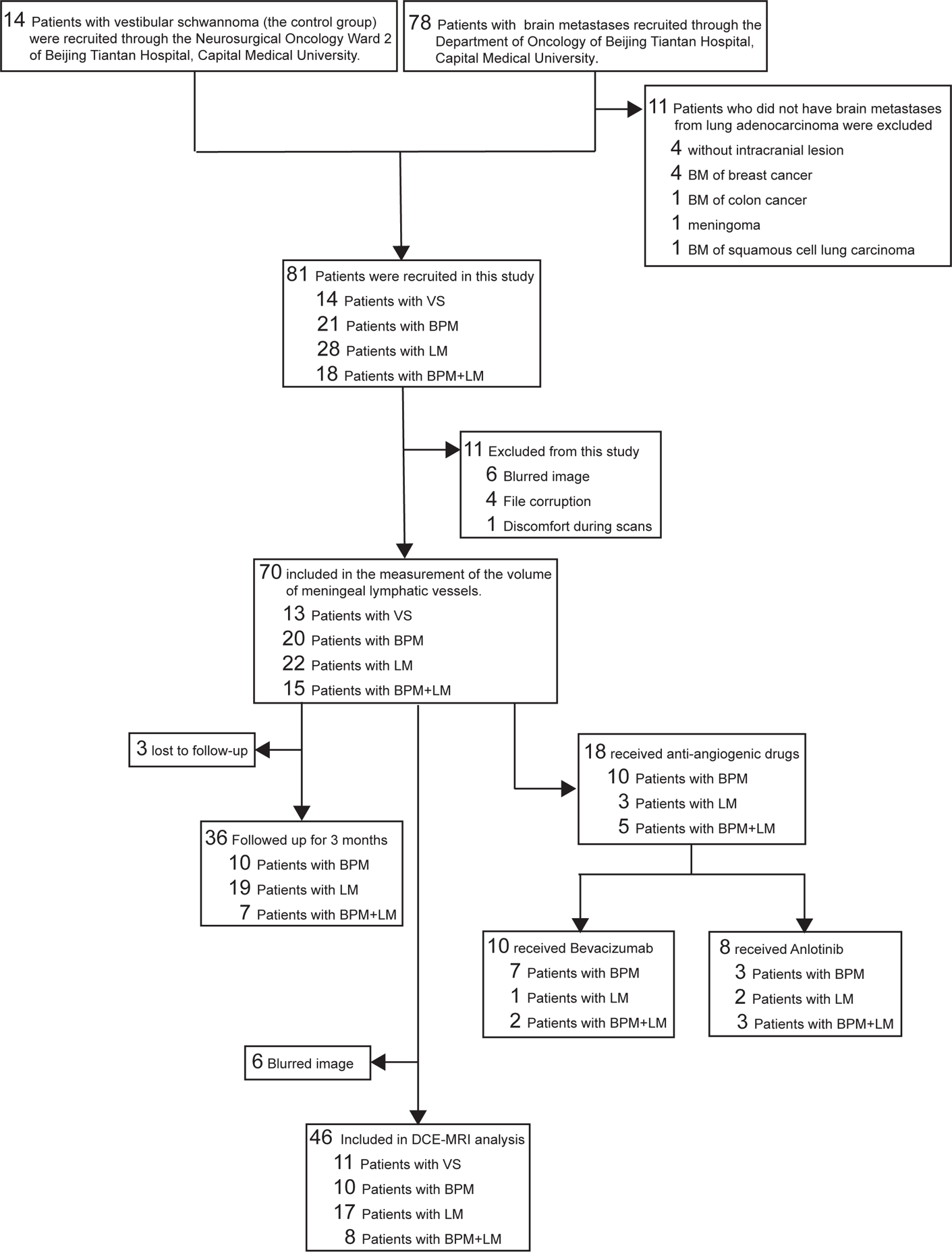


**Supplementary Figure 1** CONSORT flow diagram of participant screening and enrollment.

Figure. S2.


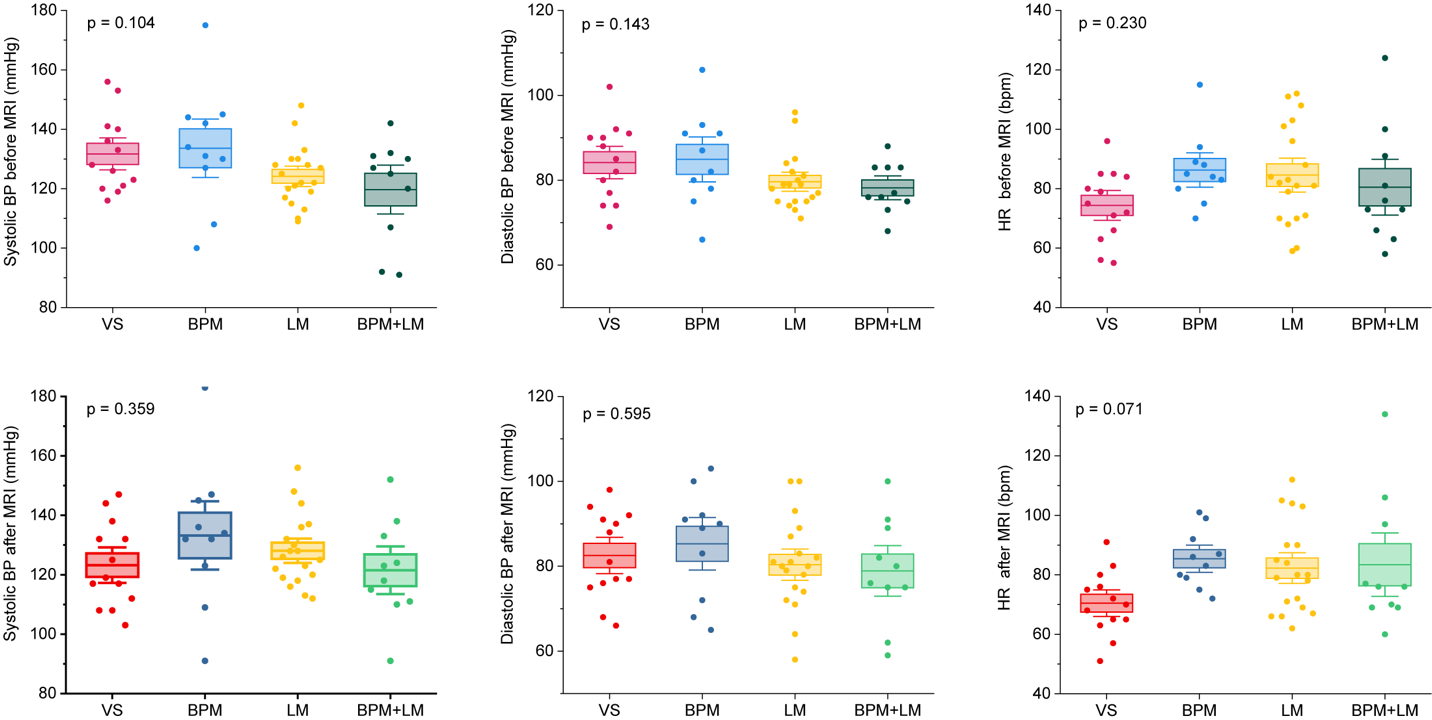


**Supplementary Figure 2** Comparison of pre-/post-MRI systolic/diastolic BP and heart rate across groups: VS (n = 13), BPM (n = 10), LM (n = 19), BPM+LM (n = 10) (one-way ANOVA with Fisher's LSD).

Figure. S3.


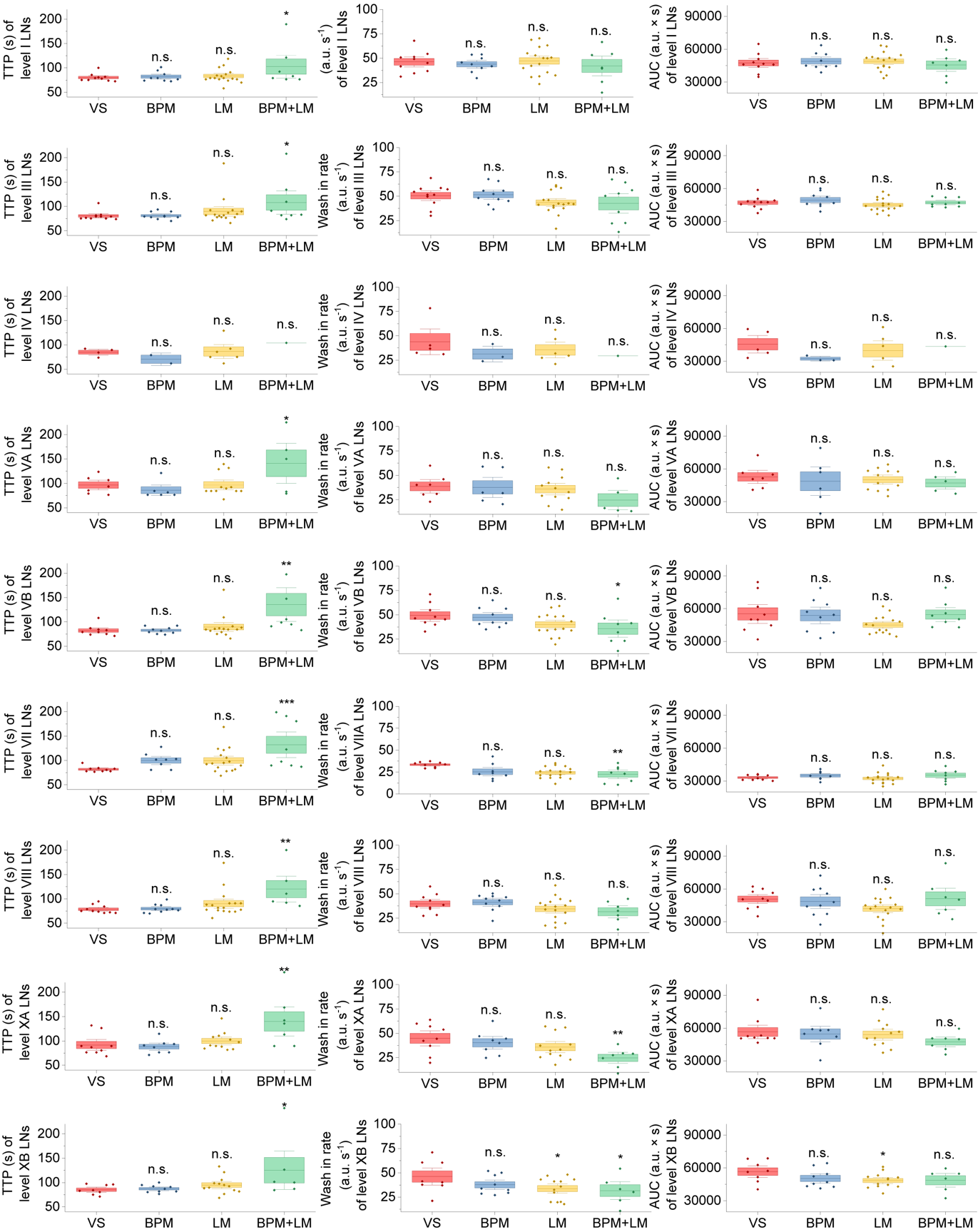


**Supplementary Figure 3** Comparison of drainage parameters (TTP/wash-in rate/AUC) in bilateral IA, III, IV, VA, VB, VIIA, VIII, XA, XB CLNs across groups: VS, BPM, LM, and BPM+LM. Variations in number of patients of each group were attributable to the inability to identify lymph nodes at the specified level on MRI. Data are mean ± s.e.m. *P < 0.05; **P < 0.01, n.s. not significant (one-way ANOVA with Fisher's LSD).

Figure. S4.


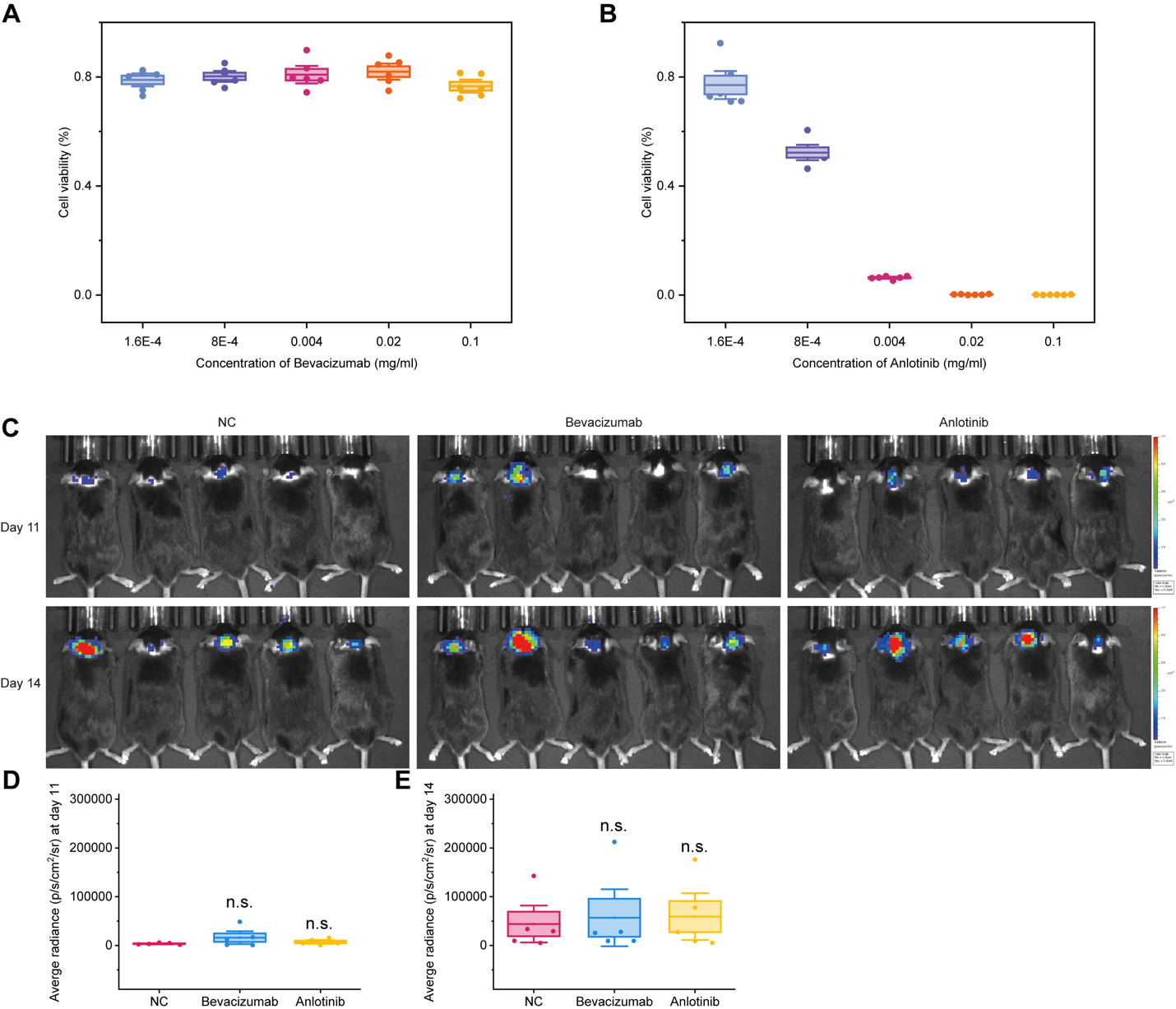


**Supplementary Figure 4** Direct cytotoxic effects of bevacizumab and anlotinib on LLC ^GFP + Luc^ cells.

(A) Cell viability under bevacizumab treatment.

(B) Cell viability under anlotinib treatment.

(C-E) Tumor growth kinetics in NC, bevacizumab, and anlotinib groups (n = 5/group). Data are presented as means ± s.e.m. n.s. not significant (one-way ANOVA with Fisher's LSD).

Figure. S5.


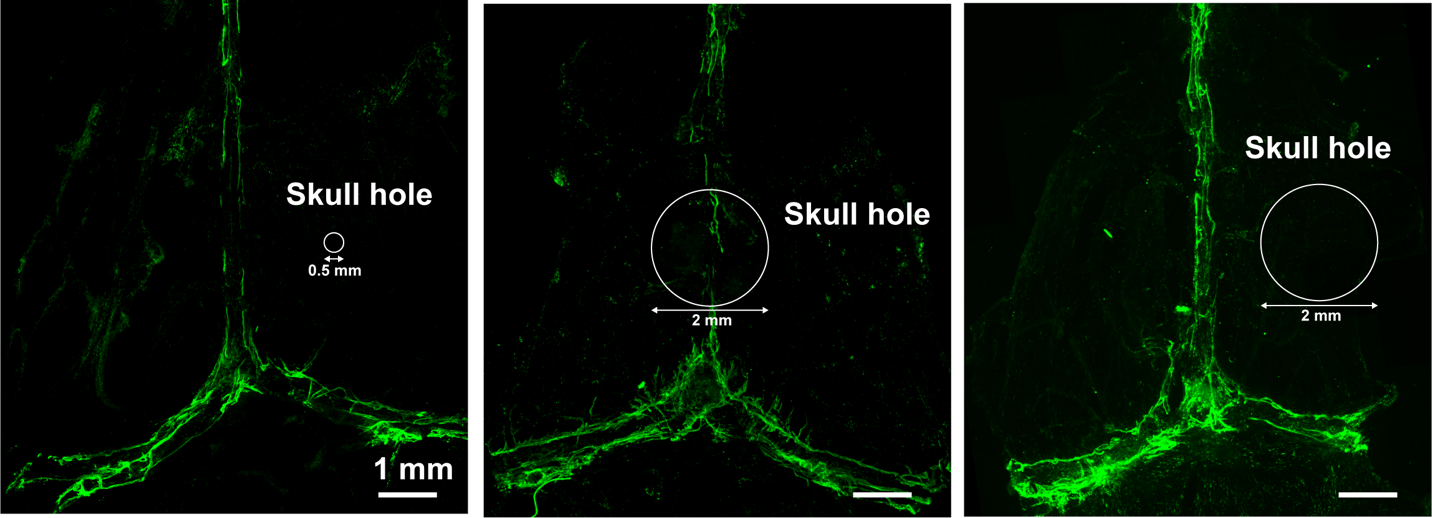


**Supplementary Figure 5** Representative LYVE-1- stained meninges 7 days post-craniectomy in C57BL/6J mice.

Figure. S6.


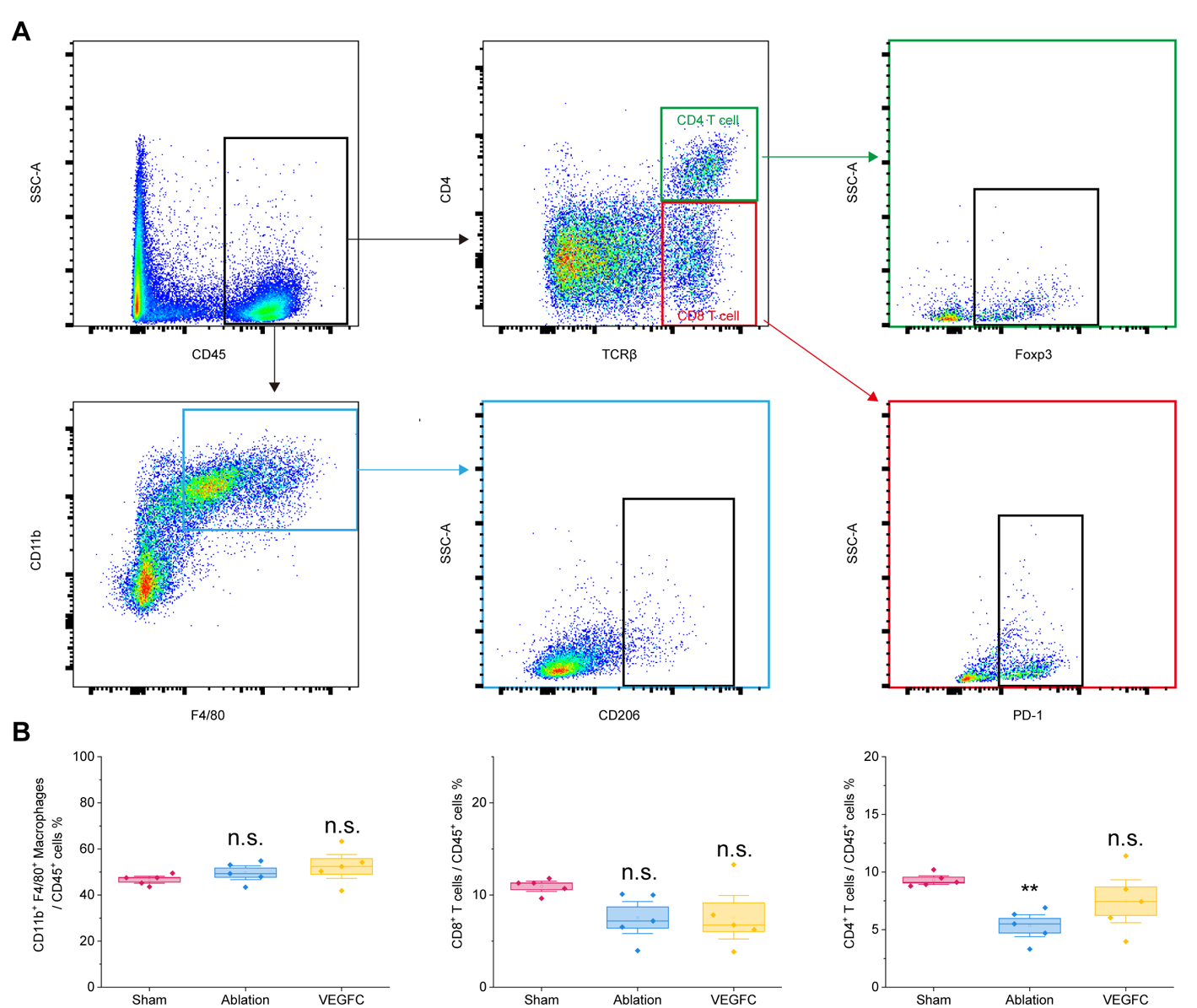


**Supplementary Figure 6** Immune response of brain tumors after ablation of dorsal mLVs or VEGFC treatment.

(A) Flow cytometry gating strategies of T cells and macrophages.

(B) Ratios of CD4^+^ T cells (left), CD8^+^ T cells (middle), and CD11b^+^ F4/80^+^ macrophages in total CD45^+^ cells in tumors. Data are presented as means ± s.e.m. **P < 0.01, n.s. not significant (one-way ANOVA with Fisher’s LSD).

Figure. S7.


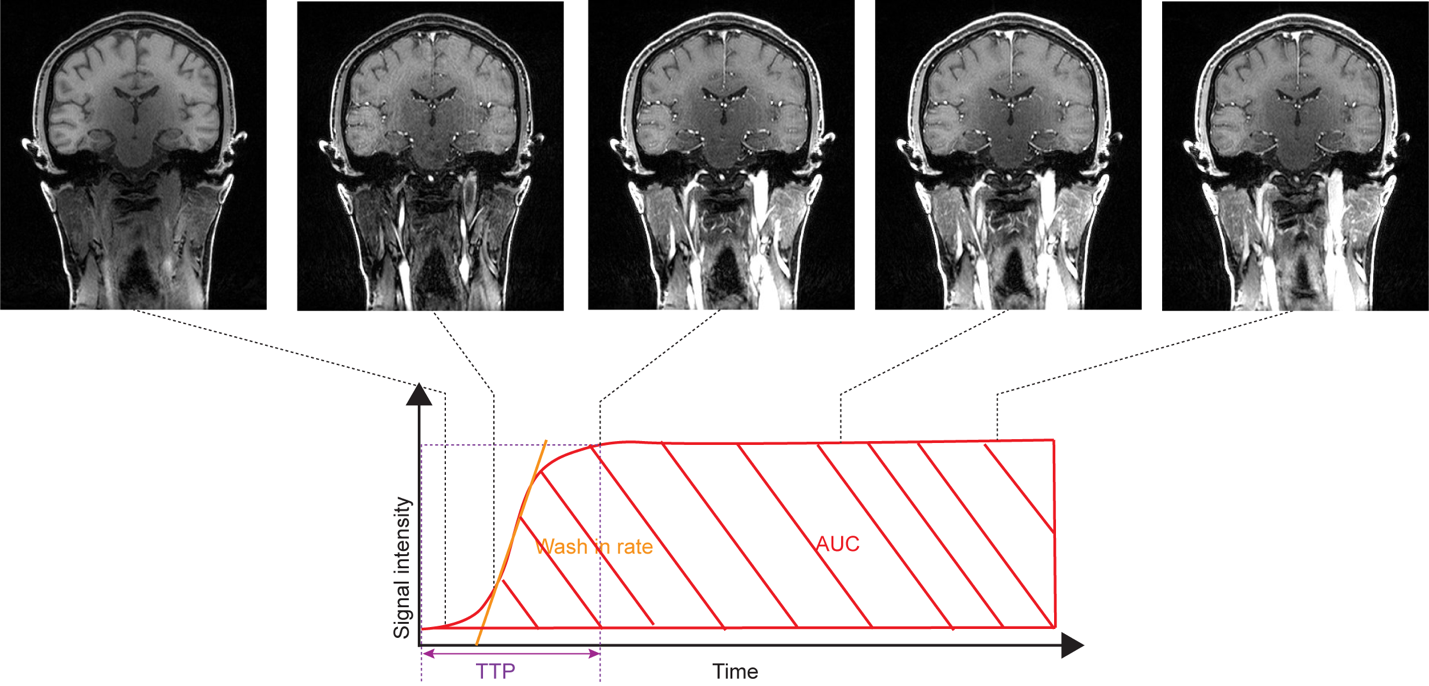


**Supplementary Figure 7** Schematic of DCE-MRI time-intensity curve parameters: Time to peak (TTP, purple) was presented as the time from start to the maximum signal intensity point. Wash-in rate (orange) was presented as the maximum slope of the time-signal intensity curve as it goes up. Area under curve (AUC, red) was presented as the area under the time-signal intensity curve after subtracting baseline.

Table S1.

| **Supplementary Table 1** Demographics and clinical characteristics of participants who underwent MRI of mLVs and CLNs. | | | | |
| --- | --- | --- | --- | --- |
|  | Vestibular schwannoma  n = 13 | Lung adenocarcinoma | | |
|  |  | BPM  n = 20 | LM  n = 22 | BPM+LM  n = 15 |
| Sex |  |  |  |  |
| Male | 7 | 10 | 10 | 6 |
| Female | 6 | 10 | 12 | 9 |
| Mean age, years | 50.4 (10.6) | 60.0 (7.2) | 55.0 (12.5) | 55.9 (9.9) |
| Anti-angiogenesis drugs |  |  |  |  |
| None | 13 | 10 | 19 | 10 |
| Bevacizumab | 0 | 7 | 1 | 2 |
| Anlotinib | 0 | 3 | 2 | 3 |
| Response after 3 months |  |  |  |  |
| Progressive | NA | 6 | 9 | 3 |
| Stable | NA | 13 | 12 | 9 |
| Loss of follow-up | NA | 1 | 1 | 3 |
| MRI-Scan |  |  |  |  |
| High resolution MRI | 13 | 20 | 22 | 15 |
| DCE-MRI | 11 | 10 | 17 | 8 |
| Abbreviation: MRI = Magnetic resonance imaging; mLVs = Meningeal lymphatic vessels; CLNs = Cervical lymph nodes; BPM = Brain parenchymal metastases; LM = Leptomeningeal metastases; DCE = Dynamic Contrast-Enhanced; NA = not applicable. Date are given as mean (s.d.). | | | | |

Table S2.

| **Supplementary table 2** Univariable and multivariable logistic regression of risk factor of disease progression within 3 months after MRI scan in LUAD brain metastasis patients who did not receive anti-angiogenic drugs (n = 36). | | | | | | |
| --- | --- | --- | --- | --- | --- | --- |
| Characteristic | Univariate analysis | | | Multivariate analysis | | |
|  | OR | 95% CI | P value | OR | 95% CI | P value |
| Age (years) | 1.075 | 0.980-1.179 | 0.125 |  |  |  |
| Sex |  |  |  |  |  |  |
| Female | Reference |  |  |  |  |  |
| Male | 1.250 | 0.299-5.230 | 0.760 |  |  |  |
| Metastatic type |  |  |  |  |  |  |
| BPM | Reference |  |  |  |  |  |
| LM | 0.643 | 0.132-3.140 | 0.585 |  |  |  |
| BPM+LM | 0.300 | 0.025-3.626 | 0.344 |  |  |  |
| Metastatic site |  |  |  |  |  |  |
| Frontal lobe (%) | 4.706 | 0.511-43.361 | 0.172 |  |  |  |
| Parietal lobe (%) | 4.154 | 0.743-23.229 | 0.105 |  |  |  |
| Temporal lobe (%) | 1.896 | 0.441-8.144 | 0.390 |  |  |  |
| Occipital lobe (%) | 4.000 | 0.849-18.836 | 0.080 |  |  |  |
| Cerebellum (%) | 0.655 | 0.157-2.724 | 0.560 |  |  |  |
| Brain stem (%) | 1.167 | 0.180-7.557 | 0.872 |  |  |  |
| Nodular enhancement |  |  |  |  |  |  |
| Negative | Reference |  |  |  |  |  |
| Positive | 1.421 | 0.238-8.478 | 0.700 |  |  |  |
| Linear enhancement |  |  |  |  |  |  |
| Negative | Reference |  |  |  |  |  |
| Positive | 0.619 | 0.144-2.659 | 0.519 |  |  |  |
| Treatment before MRI scan |  |  |  |  |  |  |
| Chemotherapy | 1.167 | 0.180-7.557 | 0.872 |  |  |  |
| Radiotherapy | 2.750 | 0.458-16.525 | 0.269 |  |  |  |
| Targeted therapy | 1.125 | 0.183-6.935 | 0.899 |  |  |  |
| Intrathecal Pemetrexed | 1.016 | 0.232-4.441 | 0.983 |  |  |  |
| Treatment after MRI scan (within 3 months) |  |  |  |  |  |  |
| Chemotherapy | 4.313 | 0.606-30.669 | 0.144 |  |  |  |
| Radiotherapy | 1.630 | 0.232-11.455 | 0.624 |  |  |  |
| Targeted therapy | 3.158 | 0.332-29.998 | 0.317 |  |  |  |
| Intrathecal Pemetrexed | 0.619 | 0.144-2.659 | 0.519 |  |  |  |
| Volume of ROI in mLVs-SSS in DIR (μm^3^) | 0.986 | 0.971-1.001 | 0.059 |  |  |  |
| Volume of ROI in mLVs-TS in DIR (μm^3^) | 1.012 | 0.988-1.037 | 0.316 |  |  |  |
| Volume of ROI in mLVs-SSS in BB (μm^3^) | 0.999 | 0.994-1.004 | 0.776 |  |  |  |
| Volume of ROI in mLVs-TS in BB (μm^3^) | 0.982 | 0.958-1.007 | 0.167 |  |  |  |
| Volume of ROI in mLVs-SSS in FLAIR (μm^3^) | 1.000 | 0.994-1.006 | 0.977 |  |  |  |
| Volume of ROI in mLVs-TS in FLAIR (μm^3^) | 0.997 | 0.978-1.017 | 0.780 |  |  |  |
| Average TTP (s) of level IIA LNs | 1.057 | 1.011-1.105 | 0.015 | 1.056 | 0.994-1.122 | 0.079 |
| Average wash-in rate (a.u. s^-1^) of level IIA LNs | 0.350 | 0.166-0.741 | 0.006 | 0.379 | 0.161-0.887 | **0.025** |
| Average AUC (a.u. × s) of level IIA LNs | 1.000 | 1.000-1.000 | 0.028 |  |  |  |
| Average TTP (s) of mLVs in DIR | 1.051 | 1.001-1.102 | 0.044 |  |  |  |
| Average wash-in rate (a.u. s^-1^) of mLVs in DIR | 0.920 | 0.848-0.999 | 0.048 |  |  |  |
| Average AUC of mLVs in BB | 1.000 | 1.000-1.000 | 0.033 |  |  |  |
| Average TTP (s) of mLVs in BB | 1.014 | 0.995-1.034 | 0.153 |  |  |  |
| Average wash-in rate (a.u. s^-1^) of mLVs in BB | 0.950 | 0.893-1.010 | 0.102 |  |  |  |
| Average AUC (a.u. × s) of mLVs in BB | 1.000 | 1.000-1.000 | 0.109 |  |  |  |
| Average TTP (s) of mLVs in FLAIR | 1.003 | 0.988-1.020 | 0.676 |  |  |  |
| Average wash-in rate (a.u. s^-1^) of mLVs in FLAIR | 0.964 | 0.904-1.028 | 0.261 |  |  |  |
| Average AUC (a.u. × s) of mLVs in FLAIR | 1.000 | 1.000-1.000 | 0.375 |  |  |  |
| Abbreviation: LUAD = lung adenocarcinoma; MRI = Magnetic resonance imaging; CI = confidence interval; OR = odds ratio; BPM = Brain parenchymal metastases; LM = Leptomeningeal metastases; SSS: superior sagittal sinus, TS: transverse sinus DIR: double inversion recovery BB: black-blood, FLAIR: fluid-attenuated inversion recovery. | | | | | | |
